# Eliciting women’s preference for prenatal testing in China: a discrete choice experiment

**DOI:** 10.1186/s12884-020-03270-7

**Published:** 2020-10-08

**Authors:** Liangzhi Wu, Yanxin Wu, Shiqian Zou, Cong Sun, Junyu Chen, Xueyan Li, Zihang Lin, Lizhi Guan, Qing Zeng, Sihan Zhao, Jingtong Liang, Rui Chen, Zhiwen Hu, Kingyan Au, Daipeng Xie, Xiaomin Xiao, Wai-kit Ming

**Affiliations:** 1 The Department of Obstetrics and Gynecology, First Affiliated Hospital of Jinan University, Guangzhou, Guangdong China; 2 The Department of Gynecology, Guangdong Second Provincial General Hospital, Guangzhou, Guangdong China; 3 Department of Obstetrics and Gynecology, The First Affiliated Hospital of Sun Yat-sen University, Guangzhou, Guangdong China; 4 School of Medicine, Jinan University, Guangzhou, Guangdong China; 5 Sun Yat-sen University Cancer Center, Guangzhou, Guangdong China; 6 Zhongshan School of Medicine, Sun Yat-sen University, Guangzhou, Guangdong China; 7 Harvard Medical School, Harvard University, Boston, MA USA

**Keywords:** Choice behavior, Women, Discrete choice experiment, Prenatal diagnosis

## Abstract

**Background:**

Pregnancy tests can be used for the early diagnosis of fetal problems and can prevent abnormal birth in pregnancies. Yet, testing preferences among Chinese women are poorly investigated.

**Methods:**

We developed a Discrete Choice Experiment with 5 attributes: test procedure, detection rate, miscarriage rate, time to wait for result, and test cost. By studying the choices that the women make in the hypothetical scenarios and comparing the attributes and levels, we can analyze the women’s preference of prenatal testing in China.

**Results:**

Ninety-two women completed the study. Respondents considered the test procedure as the most important attribute, followed by detection rate, miscarriage rate, wait time for result, and test cost, respectively. The estimated preference weight for the non-invasive procedure was 0.928 (*P* < 0.0001). All other attributes being equal, the odds of choosing a non-invasive testing procedure over an invasive one was 2.53 (95% confidence interval, 2.42–2.64; *P* < 0.001). Participants were willing to pay up to RMB$28,810 (approximately US$4610) for a non-invasive test, RMB$6061(US$970) to reduce the miscarriage rate by 1% and up to RMB$3356 (US$537) to increase the detection rate by 1%. Compared to other DCE (Discrete Choice Experiment) studies regarding Down’s syndrome screening, women in our study place relatively less emphasis on test safety.

**Conclusions:**

The present study has shown that Chinese women place more emphasis on detection rate than test safety. Chinese women place great preference on noninvasive prenatal testing, which indicate a popular need of incorporating noninvasive prenatal testing into the health insurance coverage in China. This study provided valuable evidence for the decision makers in the Chinese government.

## Background

Prenatal testing can be used for early detection of fetal abnormalities such as Down syndrome. In China, when a diagnosis of fetal Down syndrome is made, the parents can opt for continuation or termination of pregnancy. Indeed, there’re some areas where termination of pregnancy complicated with Down syndrome is not allowed, the option maybe different, hence the doctor’s consideration is even more important [1]. Several types of prenatal tests are currently used for this purpose that varies regarding diagnostic performance, invasiveness, and cost. Chorionic villus sampling that involves obtaining a sample from the placenta and amniocentesis test that requires sampling of amniotic fluid by using a hollow needle inserted into the uterus are examples of invasive testing that often provides an accurate diagnosis of potential developmental abnormalities in a fetus. Non-invasive prenatal testing (NIPT) that usually rely on a simple blood test from the mother are generally safe and less costly than invasive prenatal testing but are associated with higher false negative rates. The research found that the costs are similar as current Down’s screening at the cost of £500 per NIPT in the United Kingdom and, compared with the first trimester combined screening (measurement of serum markers PAPP-A and β-hCG as well as first-trimester ultrasound) and integrated screening (first trimester combined screening and Quad screening of serum markers (AFP, estriol, hCG, Inhibin A)), NIPT have a better Trisomy 21 detection and reduce euploid fetal loss with a lower total healthcare cost in the United States [2, 3]. Women at high risk of having babies with genetic abnormalities, usually are offered invasive prenatal diagnosis, to ensure higher detection rates. However, in approximately 1–2% of cases, invasive prenatal testing might result in miscarriage, even when the baby is healthy [4, 5]. Recently, non-invasive test, which is based on the technology to investigate the Cell-free DNA inside a maternal blood sample, has become one of the major alternatives of invasive tests and is also available in China, and the accuracy of NIPT was reported with a > 99% for trisomy 21 in both sensitivity and specificity [6–8]. However, in the past decade, it is not included in the universal coverage, patient have to pay for themselves, and the services are mainly provided by the private laboratories.

NIPT has been introduced for a self-payment option in a national health screening programme for pregnancy in some jurisdictions in China [9]. However, there is still a debate about the optimal choices, considering the trade-offs that need to be made among different attributes of various prenatal tests, including detection rate, the risk of miscarriage, invasiveness, and cost [10, 11]. Understanding women’s preferences for different attributes of prenatal tests can help physicians to enhance the process for shared decision making for the choice of best diagnostic strategy in high-risk pregnancies.

Recently, DCE has been widely used in health-care research to understand the patients’ preferences for medications and health services [12]. Lean Beulen, et al. applied DCE to estimate the preference of women and healthcare professionals for prenatal testing in Netherlands [13]. But to our knowledge, no investigations has been performed about women’s preferences for prenatal testing in China.

### Objectives

To investigate the relative importance of attributes of prenatal testing among Chinese women.

## Methods

### Study population

The study was conducted at a university hospital in South China (The First Affiliated Hospital of Sun Yat-sen University, Guangzhou, China). We recruited our sample from women with relatively high-risk pregnancy who visited the outpatient clinic for prenatal consultation between 18 January 2018 and 2 April 2018. Inclusion criteria: 1. At least 18-years-old; 2. Attending the first visit for prenatal diagnostic consultation; 3. Gestational period no more than 20 weeks; 4. Ability to comply with the protocol procedures. Exclusion criteria: 1. Women with obstetrics related medical history; 2. Those who has gestational period more than 20 weeks. Because they had previously received a prenatal diagnostic consultation and are not representative for the overall women in China.

### Discrete choice experiment

The DCE methodology is grounded in multi-attribute utility theory in economics. The technique is based on the assumption that any commodity (e.g., prenatal diagnosis test) can be characterized by several key attributes and their levels (e.g., test procedure, detection rate, test cost). Therefore, individuals choose among their options (e.g., different prenatal testing) by comparing those attributes and levels in hypothetical scenarios.

### Attribute and attribute levels

Possible attributes were identified from a panel of experienced directors of gynaecology and obstetrics department in the First Affiliated Hospital of Sun Yat-sen University. And the five attributes are finally selected as test procedure, time to wait for results, detection rate, miscarriage and test cost with their different levels. (Table 1).

**Table 1:** Attributes and Levels

Attributes	Levels of attributes (regression coding)	
**Test procedure**	L1	Invasive: requires collecting samples from amniotic fluid or placenta.
	L2	Non-invasive: only requires a sample of mother’s blood.
**Time to wait for results**	L1	1 week
	L2	2 weeks
	L3	3 weeks
**Detection rate**	L1	94%
	L2	96%
	L3	98%
	L4	100%
**Miscarriage**	L1	3%
	L2	4%
	L3	5%
**Test cost**	L1	RMB$0
	L2	RMB$2000
	L3	RMB$4000
	L4	RMB$6000
	L5	RMB$8000

### Study Design & Questionnaire

The study design and analysis followed current guidelines for conducting DCEs in a healthcare setting [14–17]. This study was a cross-sectional survey that involved completion of a questionnaire. The online questionnaire consisting of an easy-to-understand explanation of the experiment and 13 questions, which comprised of 1 demographic question and 12 choices question. The choice question has 3 options: Diagnostic test A, Diagnostic test B, and No test option, but with 3% assumed natural miscarriage rate. Table 2 shows a sample of choice question. The next 11 choice questions followed a similar format, but test profiles varied as we changed the attribute levels in each question each time and asked participants to make their choices based on the new test profiles. Using this approach, we can understand the impact of test attributes on choices that are made.

**Table 2:** A Sample Discrete Choice Experiment Choice Question

Attributes	Test A	Test B	Neither
**Test procedure**	Invasive: requires collecting samples from amniotic fluid or placenta	Non-invasive: only requires sample of mother’s blood	No test
**Time to wait for results**	3 weeks	1 weeks	
**Detection rate**	94%	100%	
**Miscarriage**	5%	3%	Natural miscarriage 3%
**Test cost**	RMB$2000	RMB$4000	No cost

Question

In Prenatal test A, B, or neither your only options, which one would you choose?

The test profiles presented in choice questions were created by generating permutations of attribute levels. There are total 360 combinations we could generate based on the level of each attribute. We then created a fractional factorial design using Sawtooth that met balance and orthogonality properties [16]. Balance (i.e., each attribute level appears equally often within an attribute) and orthogonality (i.e., each pair of levels appears equally often across all pairs of attributes) ensures minimizing the bias and improves the precision of estimated preferences. We generated 100 versions of the questionnaire and assigned each respondent to a version in a random manner to facilitate achieving balance and orthogonality. Three of 12 choice questions presented fixed scenarios that did not vary across. This include three fixed (question 1, 6 and 12) and nine random choice questions. This is a web-based questionnaire and facilitated direct data entry into our secure server [18, 19]. This questionnaire was performed using the Choice Based Conjoint application of Sawtooth Lighthouse Studio (SSI Web version 9.4.0; Sawtooth Software Inc.;) [20].

### Statistical analysis

A conditional logistic regression model was used to analyze the DCE data [21], where the choices were used as dependent variable and attribute level of tests as covariates. The levels for the 5 attributes were effects coded. The conditional logistic model provided statistical inferences about respondents’ preference weights for each of the attributes and levels included in the questionnaire. The coefficients sign (positive or negative) indicates the direction of the women’ preference for a given attribute level. To understand the trade-offs that the participants were willing to make between attributes, we calculated the marginal rate of substitution between cost and each attribute and attributed importance. The attribute importance (ranking information) was incorporated into the Mixed Logit. We explored the incorporated the ranked information by estimating the covariate explaining marginal utilities and a contraction of the marginal utility towards zero where the degree of contraction. Sawtooth Lighthouse Studio (SSI Web version 9.4.0; Sawtooth Software Inc.;)was used to perform statistical analysis.

## Results

In total 92 out of 136 women enrolled. Nineteen women (11.6%) declined to participate in the study and 25 (18.4%) did not complete all choice questions and were both excluded for analysis. The demographic information of the participants is summarized in Table 3.

**Table 3:** Demographics and Characteristics of Patients in this Study

Variable	***N*** = 92
**Age, mean (SD) years**	31.90 (5.20)
**Gestational week, mean (SD) weeks**	14.00 (5.50)
**Abnormality of a fetus of previous pregnancy (%)**	31 (33.70)
**Family history (%)**	4 (4.35)
**Abnormality of fetus history of friends or relatives (%)**	13 (14.13)

*SD* Standard deviation

Among the 92 respondents, 65 (70.7%) chose the NIPT choice for question 1 (choosing between non-invasive, invasive, and neither). The details of these options are: (1) Non-invasive, only requires a sample of the mother’s blood, 2-week time to wait for result, 98% detection rate, 3% miscarriage rate, and RMB$4000; (2) Invasive, requires collecting samples from amniotic fluid or placenta, 3-week time to wait for result, 100% detection rate, 5% miscarriage rate, and RMB$6000; and (3) No test, no cost, and 3% natural risk of miscarriage (assumed). These three choices represented the current practice, and 14 (15.2%) of the participants selected No test (Table 4). Six respondents (6.5%) chose the ‘No test’ option for all 12 questions, regardless of the attribute levels of the presented tests. Among the remaining respondents, 23 (25%) failed to choose the dominant treatment option in at least one fixed choice question (fixed choice questions 2 and 3).

**Table 4:** Response of fixed questions (*n* = 92)

Response	Fixed Question 1	Fixed Question 2	Fixed Question 3
	Current clinical practice(%)	Test B Dominant(%)	Test A Dominant(%)
**Test A**	65 (70.65)	8 (8.70)	73 (79.35)
**Test B**	13 (14.13)	73 (79.35)	8 (8.70)
**No test**	14 (15.22)	11 (11.96)	11 (11.96)

### Importance of attributes

**Fig. 1:**
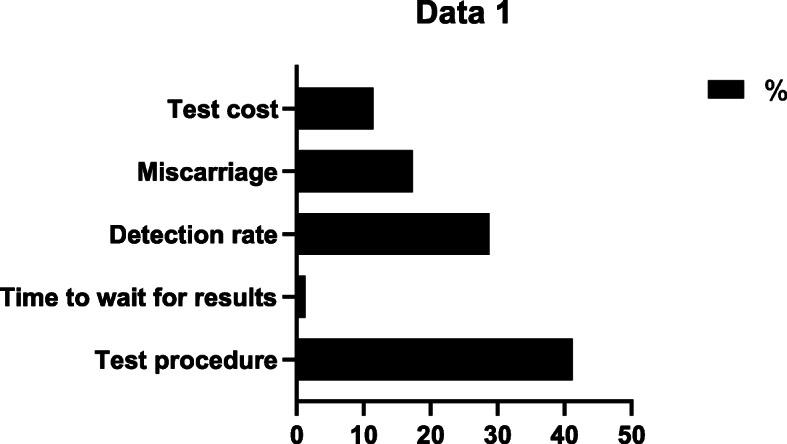
Importance of attributes

Overall, the respondents in our experiment considered the test procedure as the most important attribute, followed by detection rate, miscarriage rate, and test cost, respectively. Wait time for the results was considered the least important aspect of the test (Fig. 1).

### Conditional logit model

**Fig. 2:**
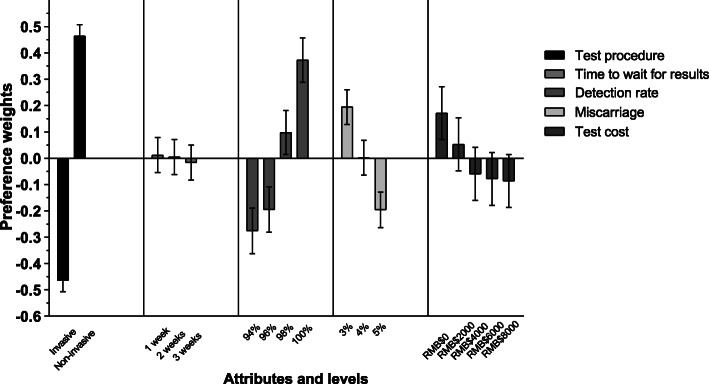
The visual presentation of estimated preference weights in the full sample

The results of the conditional logit model are concluded. (detailed in Additional file 1). *P* < 0.05 is considered to be statistically significant. Figure 2 provides a visual presentation of the estimated preference weights in our study (*n* = 92). The estimated preference weight for the non-invasive procedure was 0.928 (*P* < 0.0001). All other attributes being equal, the odds of choosing a non-invasive test procedure over an invasive one was 2.53 (95%CI 2.42–2.64). The patients also had a positive preference weight for higher levels of detection. While using a 94% detection rate as a reference group, the odds of choosing 96% was 1.085 (95%CI 0.995–1.182), 98% was 1.453 (95%CI 1.336–1.580), and 100% was 1.913 (95%CI 1.758–2.082). The negative preference weights increase for a 1-week waiting time. The odds for choosing 2-week and 3-week wait times were 0.992 (95%CI 0.929–1.061) and 0.972 (95%CI 0.909–1.039) respectively. Patients had a large negative preference related to miscarriage complication increasing. The odds of opting for an additional 1% risk of miscarriage was 0.825 (95%CI 0.772–0.881) and for an additional 2% risk was 0.677 (95%CI 0.633–0.724). There is negative preference related to a cost increase. The odds of choosing RMB$2000 was 0.888 (95%CI 0.803–0.982), RMB$4000 was 0.794 (95%CI 0.717–0.878), RMB$6000 was 0.779 (95%CI 0.704–0.861), and RMB$8000 was 0.773 (95%CI 0.699–0.854).

The coefficients signs of the test procedure and detection rate suggest that women prefer a non-invasive test with higher detection rate. The negative coefficient for time to wait for results > 2 weeks, the risk of miscarriage > 4%, and test cost > RMB $2000 indicate a preference for an earlier test, lower miscarriage rate, and lower test cost.

### Willingness to pay

Participants had a strong preference for a non-invasive test and were willing to pay up to RMB$28,810 (US$4610) for a non-invasive test. Also, women were willing to pay RMB$6061(US$970) to reduce the 1% extra miscarriage rate and up to RMB$3356(US$537) to increase 1% of the detection rate of the test. Women were less sensitive to the wait time for the result, and they were willing to pay RMB$443 (US$71) for a 1-week reduction in time to wait for results. (Table 5).

**Table 5:** Willingness to pay

Attribute	Willingness to pay	
	RMB ($)	USD ($)
**Test procedure (non-invasive)**	28,810	4610
**Time to wait for results (per 1-week reduction)**	443	71
**Detection rate (per 1% increase)**	3356	537
**Miscarriage (per 1% reduction)**	6061	970
**Test cost**	Reference	

$1 RMB = $0.16 USD

## Discussion

### Principal findings

Using a DCE, we found that participants have significant preference for non-invasive testing and high detection rate of the tests. However, compared to other attributes, participants are not sensitive to the wait time for receiving the results. Also, participants are willing to accept lower detection rate if this would imply a lower frequency of miscarriage.

## Results

Although the risk of miscarriage was not the most important concern among our participants, it had a significant impact on making choices. When compared to DCE study in Netherland regarding Down’s syndrome screening, women in both studies place preference on higher test safety, but women in our study place relatively less emphasis on test safety (i.e., miscarriage rate) [13]. This finding also differs from research from the United States in which women thought the most important feature of NIPT would be the safety of the fetus [22]. One possibility to account for these differences is that women in our cohort have a relatively higher history of abnormality of the fetus in a previous pregnancy. Our participants are also more concerned about the accuracy of the test than the miscarriage complication, meaning that for our participants, having a baby without genetic disease was more important than a procedure complication such as miscarriage. These findings might have resulted from the long-term eugenic and postnatal education that start from 1980s in China [23]. Our results show that the invasiveness of test procedure was the most influential attribute for women when they chose prenatal tests, which may attribute to the surgery pain that most participants concerned during the questionnaire collecting period. This is similar to previous study, which considered that women identified the noninvasive method of NIPT as an advantage over diagnostic tests (invasive prenatal tests) [24]. Apart from DCE, previous studies that investigated the factors affecting maternal decision on prenatal testing also reported that three dimensions predicted the intension to undergo prenatal genetic testing: the need for more scientific information, a positive attitude towards genetic testing, and the inclination to terminate pregnancy after receiving a positive test result, and women identified accuracy, early timing, testing ease, and determination of fetal sex as advantages of NIPT over other screens [24, 25].

### Clinical implications

Apart from the women preference we found in the study, we suggest that physicians’ training and communication skills on consultation are likely to be vital for the successful introduction of NIPT as the physicians could provide precise information based on the women preference. Because the women are most concerned about the test procedure and safety (extra miscarriage rate), more detailed information of the tests should be provided to the woman about the available testing options and relative advantages and disadvantages of the tests.

### Research implications

Preferences of women living in rural areas could be different and need to be explored in the future studies using larger and more representative samples. Qualitative approaches that provide an in-depth understanding of the women’s thought process and preferences regarding NIPT could complement and enhance our results using quantitative DCE approach. A further comparison study of different population groups’ preferences on prenatal testing that help in understanding the psychosocial and cultural reasons under those differences is need.

### Strengths and limitations

Our research is the first DCE study to explore the women’s preference for prenatal testing in China so far. There are some DCE studies published and examined preferences for screening and diagnostic tests, looking at attributes such as miscarriage risk [26–30]. Our study offers a systematic method to incorporate woman’ opinions in a choice of prenatal testing, which we believe can become a useful tool in patient-centered outcome research. Our estimated preference weights were robust with acceptable confidence intervals. We hope to integrate these results in the harm-benefit analysis of prenatal testing for high-risk patients. We believe that the results of this study can inform decisions about safety evaluation of current and new prenatal testing. We also believe that these results can help improve the quality of conversations about prenatal testing between physicians and women with a high-risk pregnancy.

An important advantage of using DCE is that it allows quantifying individuals’ preferences for multiple attributes, as well as the trade-offs that they are willing to make among those attributes. In this study, we collected useful information by recruiting participants from a major hospital of Guangzhou city.

Several issues, however, may limit the generalizability of our findings. Most pregnant women who took part in this study were living in the city, and our sample is not representative of the whole population of women in a similar situation in China. Our study had a small sample size. Considering we had 6 patients who chose no test for all the 12 choice questions, patients need greater guidance in their decision making. Conducting further subgroup analysis could be challenging under the given sample size. For the McFadden’s conditional logit, also known as multinomial logit [10, 29–31], we used in this paper; the strength includes this focuses on average preference, a parsimonious estimator with a unique solution, requires relatively small sample size. However, the limitations of this methodology are the assumption of homogeneity in preference. A further study on latent class analysis is needed to understand the different class preference. Our study didn’t compare the preference for prenatal testing between women and other population groups, such as the patient’s families, health professionals, etc. Only five attributes were considered for characterizing prenatal testing. However, in the real-life situation, other factors may also affect choices about prenatal tests, such as limitation of the test (such as the first moment that the prenatal testing can be performed.), false positives rate, demographic factors (social and education background) and physician consultation.

## Conclusions

Guidelines for the implementation of NIPT need to consider women’s preference to ensure patients’ need and proprieties are met as much as possible. The implementation of NIPT for routine antenatal care in China will depend on multiple factors, such as test procedure, accuracy, miscarriage risks, and costs.

Women’s strong preference for non-invasive tests demonstrates that consideration for the safety of fetus and the access to the test. Apart from this, women also strongly concern the surgery pain (test procedure) when making decisions regarding prenatal testing. This indicates the need of an effective pretest counseling and to ensure women’s better understanding of the testing process. This could lead to better-informed decisions that accommodate patient preference and values as well. Future studies conducted in larger and more representative samples are needed to enforce our current findings and to facilitate measuring potential preference heterogeneity among women.

## Supplementary information

**Additional file 1: Table A.** Estimated Relative Preference Weights.

## Data Availability

Considering the related policy about confidential patient data protection, the datasets are not available in publicly available repositories. And the datasets used or analysed during the current study are available from the corresponding author on reasonable request.

## Abbreviations

NIPT: Non-invasive prenatal testing
DCE: Discrete choice experiment
IRB: Institutional Review Board

## References

[1] Federal government backs Ohio on Down syndrome abortion law. https://abcnews.go.com/Health/wireStory/federal-government-backs-ohio-syndrome-abortion-law-68455319. Accessed 18 July 2020.

[2] Morris S, Karlsen S, Chung N, Hill M, Chitty LS (2014). Model-based analysis of costs and outcomes of non-invasive prenatal testing for Down’s syndrome using cell free fetal DNA in the UK National Health Service. PLoS One.

[3] Song K, Musci TJ, Caughey AB (2013). Clinical utility and cost of non-invasive prenatal testing with cfDNA analysis in high-risk women based on a US population. J Matern Fetal Neonatal Med.

[4] Akolekar R, Beta J, Picciarelli G, Ogilvie C, D’antonio F (2015). Procedure-related risk of miscarriage following amniocentesis and chorionic villus sampling: a systematic review and meta-analysis. Ultrasound Obstet Gynecol.

[5] Mujezinovic F, Alfirevic Z (2007). Procedure-related complications of amniocentesis and chorionic villous sampling: a systematic review. Obstet Gynecol.

[6] Lo YM, Corbetta N, Chamberlain PF, Rai V, Sargent IL, Redman CW (1997). Presence of fetal DNA in maternal plasma and serum. Lancet.

[7] Zhang H, Gao Y, Jiang F, Fu M, Yuan Y, Guo Y (2015). Non-invasive prenatal testing for trisomies 21, 18 and 13: clinical experience from 146 958 pregnancies. Ultrasound Obstet Gynecol.

[8] Gil MM, Accurti V, Santacruz B, Plana MN, Nicolaides KH (2017). Analysis of cell-free DNA in maternal blood in screening for aneuploidies: updated meta-analysis. Ultrasound Obstet Gynecol.

[9] Drury S, Mason S, McKay F, Lo K, Boustred C, Jenkins L (2016). Implementing non-invasive prenatal diagnosis (NIPT) in a National Health Service Laboratory; from dominant to recessive disorders. Adv Exp Med Biol.

[10] Brison N, Van Den Bogaert K, Dehaspe L, van den Oever JM, Janssens K, Blaumeiser B (2017). Accuracy and clinical value of maternal incidental findings during noninvasive prenatal testing for fetal aneuploidies. Genet Med.

[11] Korpi-Steiner N, Chiu RW, Chandrasekharan S, Chitty LS, Evans MI, Jackson JA (2017). Emerging considerations for noninvasive prenatal testing. Clin Chem.

[12] Clark MD, Determann D, Petrou S, Moro D, de Bekker-Grob EW (2014). Discrete choice experiments in health economics: a review of the literature. Pharmacoeconomics..

[13] Beulen L (2015). Women’s and healthcare professionals’ preferences for prenatal testing: a discrete choice experiment. Prenat Diagn.

[14] Lancsar E, Louviere J (2008). Conducting discrete choice experiments to inform healthcare decision making: a user’s guide. Pharmacoeconomics..

[15] Ryan M, Gerard K, Amaya-Amaya M (2007). Using discrete choice experiments to value health and health care: Springer Science & Business Media.

[16] Johnson FR, Lancsar E, Marshall D, Kilambi V, Mühlbacher A, Regier DA (2013). Constructing experimental designs for discrete-choice experiments: report of the ISPOR conjoint analysis experimental design good research practices task force. Value Health.

[17] Ryan M (1999). Using conjoint analysis to take account of patient preferences and go beyond health outcomes: an application to in vitro fertilisation. Soc Sci Med.

[18] Porteous T, Ryan M, Bond CM, Hannaford P (2006). Preferences for self-care or professional advice for minor illness: a discrete choice experiment. Br J Gen Pract.

[19] Guimarães C, Marra CA, Gill S (2010). A discrete choice experiment evaluation of patients’ preferences for different risk, benefit, and delivery attributes of insulin therapy for diabetes management. Patient Prefer Adherence.

[20] Margaret EK, Magdalena P, Godfrey M, Helen DP, Sandro G (2009). Women’s preferences for place of delivery in rural Tanzania: a population-based discrete choice experiment. Am J Public Health.

[21] McFadden D (2001). Economic choices. Am Econ Rev.

[22] Lewis C, Hill M, Silcock C, Daley R, Chitty LS (2014). Non-invasive prenatal testing for trisomy 21: a cross-sectional survey of service users’ views and likely uptake. BJOG..

[23] Dikotter F, Grutters J, Faas B, Feenstra L, Groenewoud H, Vugt J, Bekker M (1990). Eugenics in republican China. Republican China.

[24] Farrell RM, Mercer MB, Agatisa PK, Smith MB, Philipson E (2014). It’s more than a blood test: patients’ perspectives on noninvasive prenatal testing. J Clin Med.

[25] Pivetti M, Melotti G (2013). Prenatal genetic testing: an investigation of determining factors affecting the decision-making process. J Genet Couns.

[26] Hill M, Fisher J, Chitty LS, Morris S (2012). Women’s and health professionals’ preferences for prenatal tests for Down syndrome: a discrete choice experiment to contrast noninvasive prenatal diagnosis with current invasive tests. Genet Med.

[27] Bishop AJ, Marteau TM, Armstrong D, Chitty LS, Longworth L, Buxton MJ (2004). Women and health care professionals’ preferences for Down’s syndrome screening tests: a conjoint analysis study. BJOG Int J Obstet Gynaecol.

[28] Lewis SM, Cullinane FM, Carlin JB, Halliday JL (2006). Women’s and health professionals’ preferences for prenatal testing for Down syndrome in Australia. Aust N Z J Obstet Gynaecol.

[29] Lewis SM, Cullinane FN, Bishop AJ, Chitty LS, Marteau TM, Halliday JL (2006). A comparison of Australian and UK obstetricians’ and midwives’ preferences for screening tests for Down syndrome. Prenat Diagn.

[30] Ryan M, Diack J, Watson V, Smith N (2005). Rapid prenatal diagnostic testing for Down syndrome only or longer wait for full karyotype: the views of pregnant women. Prenat Diagn.

[31] Chan YM, Sahota DS, Leung TY, Choy KW, Chan OK, Lau TK (2009). Chinese women’s preferences for prenatal diagnostic procedure and their willingness to trade between procedures. Prenat Diagn.

