# Supplementary material for: Eliciting women’s preference for prenatal testing in China: a discrete choice experiment
Source: BMC Pregnancy Childbirth. 2020 Oct 8;20:604. doi: 10.1186/s12884-020-03270-7 (PMC7542883; doi:10.1186/s12884-020-03270-7)
Supplement: Supplementary file 1 — Additional file 1: Table A. Estimated Relative Preference Weights. [file 12884_2020_3270_MOESM1_ESM.docx]

**Table A.** Estimated Relative Preference Weights

|  | **Estimated preference weights** | | | | **Odd ratio** | |
| --- | --- | --- | --- | --- | --- | --- |
| **Attribute** | **Level** | **Coefficient** | **SE** | **P value** | **Odd ratio** | **95% CI** |
| **Test Procedure** | **Invasive: requires collecting samples from amniotic fluid or placenta** | -0.464 | 0.043 | <0.001 | Reference |  |
|  | **Non-invasive: only requires sample of mother’s blood** | 0.464 | 0.043 | <0.001 | 2.531 | (2.424 - 2.642) |
| **Time to wait for results** | **1 week** | 0.012 | 0.066 | 0.856 | Reference |  |
|  | **2 weeks** | 0.004 | 0.066 | 0.946 | 0.992 | (0.929 - 1.061) |
|  | **3 weeks** | -0.017 | 0.066 | 0.804 | 0.972 | (0.909 - 1.039) |
| **Detection rate** | **94%** | -0.276 | 0.087 | 0.002 | Reference |  |
|  | **96%** | -0.195 | 0.086 | 0.026 | 1.085 | (0.995 - 1.182) |
|  | **98%** | 0.098 | 0.084 | 0.246 | 1.453 | (1.336 - 1.580) |
|  | **100%** | 0.373 | 0.085 | 0.004 | 1.913 | (1.758 - 2.082) |
| **Miscarriage** | **3%** | 0.194 | 0.066 | 0.978 | Reference |  |
|  | **4%** | 0.002 | 0.066 | 0.004 | 0.825 | (0.772 - 0.881) |
|  | **5%** | -0.196 | 0.067 | <0.001 | 0.677 | (0.633 - 0.724) |
| **Test cost** | **RMB$0** | 0.172 | 0.100 | 0.091 | Reference |  |
|  | **RMB$2000** | 0.053 | 0.101 | 0.601 | 0.888 | (0.803 - 0.982) |
|  | **RMB$4000** | -0.060 | 0.101 | 0.558 | 0.794 | (0.717 - 0.878) |
|  | **RMB$6000** | -0.079 | 0.100 | 0.436 | 0.779 | (0.704 - 0.861) |
|  | **RMB$8000** | -0.086 | 0.100 | 0.392 | 0.773 | (0.699 - 0.854) |
| **Log likelihood** | | | -757 | | | |
| **Log likelihood of model without predictors** | | | -910 | | | |
| **Akaike Info Criterion (AIC)** | | | 1539 | | | |
| **Bayesian Information Criterion (BIC)** | | | 1600 | | | |

SE: standard error
